# Introduction of a collaborative quality improvement program in the French cystic fibrosis network: the PHARE-M initiative

**DOI:** 10.1186/s13023-017-0745-7

**Published:** 2018-02-08

**Authors:** Dominique Pougheon Bertrand, Guy Minguet, Pierre Lombrail, Gilles Rault

**Affiliations:** 1 LEPS EA3412, Sorbonne Paris Cité University, Bobigny, France; 2 Mines-Nantes School, Nantes, France; 3 Cystic Fibrosis Center, Fondation Ildys, Roscoff, France

**Keywords:** Cystic fibrosis, Quality improvement program, Clinical microsystem, Learning and leadership collaborative, Rare disease, Patient registry

## Abstract

**Background:**

An agreement, signed in 2007 by the 49 French Cystic Fibrosis Centers, included a commitment to participate, within the next 5 years, in a care quality assessment and improvement program (QIP). The objective was to roll out in the French Cystic Fibrosis (CF) care network a QIP adapted from the US program for Accelerating Improvement in Cystic Fibrosis Care developed by The Dartmouth Institute Microsystem Academy (TDIMA) and customized by the US CF Foundation between 2002 and 2013.

**Methods:**

The French national team at the Nantes-Roscoff CF Center of Expertise was trained at TDIMA and visited US CF centers involved in US Learning and Leadership Collaboratives (LLCs). It introduced the PHARE-M QIP in France by transposing the Action Guide and material. A PHARE-M LLC1 including seven centers, underwent two external assessments. Adjustments were made, then a PHARE-M LLC2 was rolled out at seven more centers in two regions. On-site coaching was strengthened. The teams’ satisfaction was assessed and further adjustments were made. In 2014, the program sought recognition as a continuing education program for healthcare professionals.

**Results:**

Ninety-six trainees including 14 patients/parents from the 14 CFCs volunteered to participate, test and adapt the program during LLC1 and LLC2 sessions. Comparison of patient outcomes collected in the Registry report by CF center, reflection on potential best practices, selection by each team of an improvement theme, implementation of improvement actions, and exchanges between teams fostered the adhesion of the teams. The program strengthened quality of care, interdisciplinary functioning and collaboration with patients/parents at the centers. The satisfaction expressed by the teams increased over time. A post-PHARE-M cycle maintains the focus on continuous quality improvement (CQI). In 2015, PHARE-M was recognized as a continuing professional development program in healthcare.

**Conclusions:**

The PHARE-M is a complex intervention in multidisciplinary teams working in a variety of hospital settings. A confluence of factors motivated teams to engage in the program. Involving Patient/Parent in quality improvement (QI) work and developing patient therapeutic education for self-management appeared to be complementary approaches to improve care. Incorporating the program into hospital continuing education insures its sustainability. Transparency of Patient Registry indicators per center published in a brief lapse of time is required to effectively support CQI. The impact of the PHARE-M on patient outcomes after 3 years is the subject of a research program funded by the French Ministry of Health whose results will be available in 2017.

## Background

The follow-up of cystic fibrosis (CF) patients in specialized care centers has been shown as an independent factor for patients better outcomes and longer survival in patients [1, 2]. In the 21st century Quality Improvement Programs (QIPs) have emerged as new strategies to reduce variability of care and facilitate the implementation of best practices across centers. Following the publication in 2001 of the report entitled *Crossing the Quality Chasm* [3], the US CF Foundation (US CFF) launched a benchmarking study to analyze the differences in patient outcomes across the CF care network. This study highlighted differences in median survival between the 10 best centers and all other centers. The decision was made to design and implement Learning and Leadership Collaboratives (LLCs) with an overarching goal of delivering the best possible care to all patients and improving clinical outcomes [4]. This program was developed by the Dartmouth Institute Microsystem Academy (TDIMA) [5], then adapted, tested and implemented into the CF network starting in 2002 [6].

The cystic fibrosis care center network in France was formalized in 2002, following generalization of systematic newborn screening for CF, to deliver specialized CF care from the diagnosis to adulthood [7]. In 2006, the French National Authority Health published a CF Diagnosis and Treatment Protocol for CF [8]. The French National CF Observatory, modelled on the CF American Patient Registry questionnaire, was established in 1992. Its objective has evolved into taking a comprehensive census of the population [9]. It is now known as the French CF Registry [10] and was certified by the French National Committee of Rare-Diseases Registries in 2007. It is fed into the European CF Registry and contributes to European epidemiologic studies [11]. Within the framework of the first French National Plan for Rare Diseases, the French Ministry of Health designated two CF Centers of Expertise in 2006 to carry out national action plans across the CF care network. The Nantes-Roscoff Center of Expertise action plan featured the following priorities: health information and communication systems, therapeutic patient education, clinical research in the social sciences and transplantation, and a care QIP. An agreement prepared in 2007 and signed by the heads of all CF centers included a commitment to “*participate, within the next five years, in a care quality assessment and improvement program to be offered by the Centers of Expertise in collaboration with the French CF Society, the French Ministry of Health and patient organizations*.”.

Since 2006, communications at the North American CF Conference and the European CF Conference have reported successful experiences on the part of centers engaged in the US CF LLCs. At a conference in France in 2008 by the French CF patient organization Vaincre la Mucoviscidose and the French CF Society, results of the US LLCs on CF care and patient outcomes were presented to an assembly of clinicians, care providers, patients and parents. A working group including representatives of the patient organization and of the Nantes-Roscoff EC was formed to reflect on a method for developing and implementing a QIP in France inspired from the US CF QIP. With the support of the CF Foundation, a training for the lead physician of the Nantes-Roscoff EC at The Dartmouth Institute as well as visits to centers engaged in the US CF QIP were organized in 2008. These confirmed the interest of transposing this program to France in order to benefit from this experience and reduce the time taken to develop a QIP in France [12]. A team including a parent (an engineer by training) and a physiotherapist was formed at the Nantes-Roscoff Center of Expertise. A presentation by the US QIP coordinator at the Vaincre la Mucoviscidose General Assembly (Reims 2011) was made to inform the French CF community of the importance and feasibility of such a QIP in CF care in France. Both the physiotherapist and the parent went to TDIMA for training and to US centers engaged in LLCs to observe the results achieved following the implementation of a QIP. This was made possible by a grant from the patient organization. Under the supervision of experts from Dartmouth and the CFF, the French team began the translation of the CF Action Guide and educational tools, registered on the Dartmouth CF network’s collaborative website, and reflected on the resources needed to implement the program in France. When the program started in France in 2011, some differences between the two countries, such as certain characteristics of the French healthcare system and unique features of the French CF care model and the French cultural context, questioned the success of transposition of the program, the adherence by stakeholders and the achievement of results on the level reported by the United States.

The aim of this article is to report and reflect on the experience of introducing the PHARE-M^*^ QIP in France, between 2011 and 2015, through two annual LLCs leading to the standardization of the final program as a continuing professional development training program on the French hospital continuing education website. We present the factors that gained the teams’ adherence, the synergies at work and the adaptations that led to the adoption of the program in the French CF network. Based on our experience, we discuss the elements that we believe to be essential in transposing this CF LLC QIP to the context of another country, since the European CF Society have paved the way for care quality improvement initiatives across the CF care center network in Europe.

## Methods

This QIP, designed according to the systematic approach described by Nelson, Batalden, and Godfrey [13], is focused on the clinical microsystem, which includes the multidisciplinary care team, patients and their family. The LLC QI format has been adopted by the CF Foundation in 2002 *to support the* CF *centers’ work to reduce the variation in patient outcomes across the US network*. *This adoption included adaptations to the specificities of the care center network, such as local culture, patient population and multidisciplinary staff and the healthcare system in which it existed,* as described by Godfrey and Oliver [6]. The French program is derived from the 2006 US LLC program and benefitted from the experience with and customization of the program in the US CF care network.

### French national team responsible for transposing of the US CF LLC

A French national team was formed comprising the lead physician at the Nantes-Roscoff Center of Expertise, his assistant, a parent of an adolescent with CF (an engineer by training), a physiotherapist and the head of information and communication system projects. The physician, physiotherapist and parent had been trained in a quality course at TDIMA, and had visited several CF centers involved in the CF LLCs for years [12]. The physician in charge of the French national therapeutic patient education program (TPE) and director of the pediatric CF center in Nantes, was closely associated with the team and led its testing at her center. This team is hereinafter referred to as the “national team”. Due to its composition, the national team included two main features unique to French CF model of care: 1) the CF therapeutic patient education program, validated in 2005 by the French health authorities and structured according to developmental stages in children and needs in terms of management of complication in adults (http://etp.centre-reference-muco-nantes.fr), and 2) respiratory physiotherapy care, delivered to patients at home according to the French National Diagnosis and Treatment Protocol and reimbursed by the French national health insurance system. The national team also strongly emphasized the involvement of patients and parents in the QIP at each center. A recruitment procedure was put in place to identify in the patient caseload at each center individuals with CF or parents of children with CF who were motivated, available, at ease in their relationships with professionals, capable of self-expression in a group, able to communicate via Internet with the team. The patient or parent was enlisted as a full member of the local quality improvement team and their travel expenses were reimbursed by the patient organization Vaincre la Mucoviscidose.

### Transposition of the US CF LLC into a first version of the PHARE-M LLC

Training materials were provided free of charge by the US CFF and access to TDIMA’s electronic resources was authorized. Resources were developed before the program started in France (September 2011). They included:

- the translation of training materials, including the Action Guide for Accelerating Improvement in Cystic Fibrosis Care [14] under a Dartmouth Director supervision;
- the drafting of a French national report entitled “Registry, a Tool for Quality Improvement” (RTQI), to inform patients and parents and present the usefulness of the French CF Registry to assess improvement on patient outcomes; “The 10 Goals of the PHARE-M” (see below) and an itemization of each goal with the respective roles in a for care improvement partnership to be played by the patients, their family and the healthcare providers;
- the creation of a website dedicated to the PHARE-M (http://pharem.centre-reference-muco-nantes.fr/) containing tools, training materials and updates and serving as a messaging tool dedicated to the teams engaged in the PHARE-M; and
- the selection of a web conference tool for remote training meetings.

The 10 Goals of the PHARE-M are:

1. Parents and patients are full partners of the healthcare team. Each patient/family has a right to clear and understandable information.
2. Each patient, regardless of his or her geographical, social, and cultural circumstances, enjoys effective multidisciplinary care.
3. Each patient/family has a right to therapeutic education to aid in acquiring or strengthening the skills required to best manage life with cystic fibrosis.
4. Patients grow normally and have a normal nutritional status.
5. Respiratory infections and exacerbations thereof are detected as early as possible, and appropriate treatments are started without delay.
6. Physical and sports activities are encouraged from an early age and adapted to each patient throughout his or her life.
7. Suitable measures are put in place and hygiene advice is given to prevent cross-contamination.
8. Complications, including diabetes, are diagnosed and treated early.
9. All patients who progress to a state of severe respiratory failure are informed of their therapeutic alternatives, then either supported in their decision to undergo transplantation or accompanied at the end of life.
10. Post-transplant care aims at sustainable improvement in quality of life and in physical, psychological, and social health.

### The Pilot PHARE-M LLC1 (September 2011 – June 2012)

The PHARE-M LLC1 enrolled 7 volunteer centres, including four CF centers from the two national French national Centers of Expertise of Nantes-Roscoff and Lyon, thanks to close professional networking. A multidisciplinary “quality improvement team” was formed at each center included a physician leader, four to five professionals and a parent or a patient. Vaincre la Mucoviscidose agreed to reimburse the travel fees of the teams – including those of the patients/parents – and give each center a grant covering a 0,20 FTEs for a nurse for 1 year, corresponding to the extra time required for data analysis and teamwork management.

Four Face-to-face LLC meetings were organized. At these meetings, theoretical presentations of the method illustrated with examples drawn from the American teams were alternated with practical exercises by the French center teams. Each team analyzed its patient outcomes and selected a theme for improvement for a target patient population. Patient data was available for each center from the 2009 Patient Registry report by center; however, some indicators presented weaknesses such as body mass index (BMI) being expressed for children as an absolute value and not as a percentile or Z-score. This forced the teams to collect specific data from their patient electronic records. The teams were offered Action Guide tools (satisfaction surveys, activity analysis grids, communication tools, etc.) and took advantage of the opportunity to adapt them to their setting. International experiences published in the literature were presented [15, 16] and the teams were reminded of CF care guidelines [17]. Each team identified actions to redesign its processes, in line with its theme for improvement, to be tested according to successive PDSA cycles. The teams’ satisfaction and suggestions were recorded at each meeting and an overall score was displayed on the PHARE-M website.

Close collaboration with the TDIMA and the CFF was sustained over the course of LLC1 through:

- the participation of members of the national team, as well as physicians at several pilot centers, in the adult LLC session at the North American conference in Anaheim (October 2011);
- the participation of the Director of TDIMA Clinical Microsystem Group in the third face-to-face meeting to supervise the poster session meeting (PHARE-M LLC1, Marseille, March 2012);
- the trainings for the physiotherapist and the parent on the national team in the TDIMA’s “eCoach the Coaches” course at the same time as the PHARE-M LLC1.

### Assessments of the pilot PHARE-M LLC1

The PHARE-M being an innovative approach to QI in France, some key stakeholders were dubious as to its applicability in the French CF care network. The head of the Nantes-Roscoff Center of Expertise asked a Mines-Nantes School sociological researcher to perform a first assessment of the program to analyse the factors for its success and barriers to its adoption, and the patient organization asked a consulting a firm to perform a second assessment to inform its decision as to whether to continue to fund the program.

The first assessment took place during LLC1. The assessor participated as an observer during two web meetings and the third Face-to-Face meeting. The assessment included familiarization with PHARE-M documents, interviews with a panel of professionals and patients/parents on the quality improvement teams, an interview with the members of the national team, an interview with the Director of TDIMA, and a visit to one site. All interviews and focus groups were recorded and fully transcribed. The data was exploited (coding, categorization), processed (analysis, validity) and interpreted according to the standard thematic content analysis protocol (Miles & Huberman [18]). This was followed by manual grouping and counting within an analysis framework with the following dimensions: process applicability (terminology, formalization, tools, distance web meetings); incorporation of patients and parents (roles, time spent, barriers); national/regional coordination (roles, nature of support, incorporation mechanisms); process adoption (perceived benefits and costs, working atmosphere, engagement, acquisitions); and impact (operation, working practices, cooperation with the stakeholders). The report was submitted in July 2012 for consideration to adjust the PHARE-M LLC2.

The second assessment was contracted at the end of LLC1 to evaluate the effectiveness of this QI method in France, and to perform a comparative analysis between aims and outcomes achieved (efficiency) and between actions performed and expenses (efficacy). The study methodology included: familiarization with the PHARE-M documents and the literature on CF (French National Diagnosis and Treatment Protocol, French National Registry, etc.); investigations into four engaged CFC sites (Versailles, Lyon pediatric, Reims, and Roscoff) with professionals and patients/parents; telephone interviews with the members of the national team and patients/parents. The report was submitted during the October 2012 meeting of the board of directors of the patient organization, and the decision as to whether to continue funding was voted on in December 2012.

### Main adjustments in the PHARE-M LLC2

Following these two assessments, the national team made adjustments to the program, thus further customizing the second version of the PHARE-M (see below). The patient organization continued to fund the travel fees of the teams and the extra-time worked by a referent professional on the team at each center. No funding was allocated to the national team for intensive coaching of the teams at each center.

The main adaptations in the PHARE-M LLC2 were:

1. Drafting of a second version of the Action Guide illustrated with examples from the French teams in LLC1 instead of examples borrowed from the American teams;
2. Reduction of certain theoretical presentations in the training materials in favor of more exercises during face-to-face meetings;
3. Updated and revised version of the RTQI with was more systematically offered to patients/parents and professionals, either in its entirety or as separate chapters focusing on the goal chosen by the team at the center;
4. Formalization of the “PHARE-M referent” role on each quality improvement team, for a non-physician professional subsidized by the patient organization;
5. Incentive to enlist a quality engineer from the hospital quality department on the quality improvement team at the center, this professional sometimes becoming the PHARE-M referent;
6. One on-site coaching of the team at each center, offered during a visit by the program coordinator and focusing on mapping the clinic process with the “Shadowing a Patient” method [19]; and
7. Simplification of the PHARE-M website by withdrawing the PHARE-M specific messaging tool for the teams engaged in the PHARE-M as they did not use it in addition to their existing messaging tool.

### Inter-regional rollout of the PHARE-M LLC2 (September 2012 – June 2013)

A second PHARE-M LLC session was planned to enroll the centers in the two French inter-regions of Rhône-Alpes-Auvergne and Grand-Ouest belonging to the regional care network of the two CF Centers of Expertise of Nantes-Roscoff and Lyon that could not have been included in the first session.

The teams’ satisfaction and suggestions were recorded at every face-to-face meeting and web conference during LLC2. They led to two more adjustments to the training material:

- rearrangement of the content of the third and fourth face-to-face sessions by moving up the benchmarking visit and delaying the poster at the end of the LLC session; and
- strengthening of the link with TPE, underlying the importance of programming time for educational sessions during the clinic visit, focusing on the improvement goal and particular needs of the patient.

The teams also requested that a “post-PHARE-M cycle” be established to maintain a focus on quality improvement and have CFCs continue to exchange experiences after the LLC until they achieved their goal for improvement (two to 3 years after the training year). This was discussed with the patient organization for purposes of obtaining additional funding to organize an annual CQI meeting at a CF center for benchmarking and sustaining QI work.

### Standardization and sustainability of the PHARE-M

The growing difficulty of enlisting new CFCs and the risk of jeopardizing patient organization funding led the national team to conceive of different avenues for perpetuating the PHARE-M and its rollout throughout the CF network.

First, a research project was drawn up in an attempt to respond to the recurrent request for evidence of the PHARE-M’s positive impact on patient outcomes. The PHARE-M Performance project was submitted at a call for projects by the French Ministry of Health in February 2012. The project was selected by the Ministry on 5 December 2012 and funded for a three-year study. Its protocol combined a quasi-experimental evaluation of the effectiveness of the program to change patient outcomes over the course of 3 years with a process evaluation [20]. Following a realistic approach, the latter was designed to understand what works, for whom and under which circumstances (context) [21]. The success of the PHARE-M performance project at this call for projects was seen as a means to give credibility and recognition to the PHARE-M as well as funding to the national team for further interventional research.

Second, systematic efforts were made to incorporate the PHARE-M’s into hospital accreditation process. The announcement of certain professional practice evaluation (EPP) actions for improvement and the participation of a hospital quality engineer on the quality improvement team at several centers were actively sought to improve the acceptability of the program in hospitals alongside more traditional certification methods.

Finally, continuing professional development in the field of hospital continuing education, which started in 2013 [22–24], offered an opportunity to standardize the PHARE-M into a hospital continuing education program without modifying its content or curriculum except to have it take place during a calendar year (January through December). Recognition by the hospital continuing education authority of the PHARE-M as a CPD program was sought as it was key to further roll-out.

## Results

### Results of PHARE-M LLC1 & LLC2

Seven centers volunteered to test and propose improvements to the program in the PHARE-M LLC1: four pediatric centers (Lyon, Nantes, Paris Robert Debré, and Versailles), one adult CFC (Lyon), and two pediatric teams at mixed centers (Reims and Roscoff) following up a total of about 1200 patients out of the 6500 patients in the Registry in 2011. Seven more centers from the two French inter-regions of Rhône-Alpes-Auvergne and Grand-Ouest engaged in the PHARE-M LLC2: three pediatric centers (Angers, Grenoble, and Rennes), two adult centers (Nantes and Rennes), and two mixed centers (Clermont-Ferrand and Morbihan), to which the adult team at the Roscoff center was added, following up about 800 more patients.

Ninety-six trainees from the 14 CFCs participated in the two annual PHARE-M sessions. More than half of the participants (54%) belonged to the multidisciplinary “core” team and 15% were patients or parents of patients. Healthcare providers on the quality improvement teams represented a total of 75 people, patients/parents represented 15 people, and non-healthcare professionals represented six people. Psychologists and dieticians were particularly strongly enlisted to the quality improvement teams (9/75 (12%) and 7/75 (9.3%) respectively).

Among those 14 centers (out of 45 CF care centers in France), three elected a theme for improvement related to adult care, one chose a theme related to transition to transplantation, one chose a theme related to transition to adult care, and nine chose a theme related to either respiratory or nutritional pediatric care. Four of them worked closely with the Quality Department at their hospital. Companion articles in this supplement present the changes in processes and clinical outcomes achieved in some centers between 2012 and 2015 and the links developed between the program and the general quality process at the hospital [25–27]. They show that working in QI has allowed these teams to achieve their goals and even exceed them on various themes of improvement such as FEV1 for adolescents, BMI for children 2 to 12 y.o. or time on the lung transplant waiting list. The statistical analysis of the PHARE-M Performance research project, which will assess the effectiveness of the program to change patient outcomes at centers involved in LLC1 & 2, will be performed on the Registry data from 2011 to year 2015 and results will be available by the end of 2017.

The assessment of the teams’ satisfaction showed an increase between LLC1 and LLC2, as expressed at each training meeting and for the LLC overall, reflected in the median of all the participants’ scores on a scale from 0 to 10, where 10 represented maximum satisfaction (median score = 7.48) and the LLC2 (median score = 8.16).

The final PHARE-M curriculum is presented in Table 1.

**Table 1:** Final PHARE-M curriculum

Phase	Activity: 44 h, 32 h face-to-face meetings, 8 h web conf. ESE: expertise and sharing of experience face-to-face meeting Web Conf.: remote conference organized via internet PDSA: plan-do-study-act
Phase 1: Organization of the quality improvement teams at the centres	Information meeting on the PHARE-M
	Organization of the quality improvement teams at the CFCs and enrollment in continuing education
	*Web conf.: progress report on the preparatory phase*
Phase 2: Analysis of the clinical microsystem	ESE1: Presentation of the methodology and analysis tools (5Ps) and initialization of the analyses in practice
	Analysis of the clinical microsystem by the quality improvement team at the CFC
	*Web conf.: progress report on the analyses at the CFCs*
Phase 3: Planning of the actions for improvement in the clinical microsystem	ESE2: Presentation of the results of the analyses, selection of the themes for improvement and quantified objectives, examination of the ideas for change and foreshadowing of the actions for improvements (PDSA cycles)
	Organization of the actions and preparation of the PDSA cycles
	*Web conf.: progress report on the definition of the PDSA cycles*
Phase 4: Implementation of the actions for improvement according to the PDSA cycles and measurement of the outcomes	ESE3: Benchmarking visit, incorporation of best practices into the actions for improvement, and review of the schedules for implementation of the PDSA cycles
	Implementation of the first PDSA cycles and operational measurement indicators
	*Web conf.: progress report on the implementation of PDSA cycles*
	ESE4: Presentation of the teams’ posters and presentations

At the teams’ request, two post-PHARE-M cycles were offered in 2014, one pediatric and the other adult, consisting of one meeting per year at a CFC, including a benchmarking visit, an account of the progress and outcomes of the teams’ actions, exchanges between the teams, and reminders fundamental aspects of the QIP.

Thirteen teams prepared their poster at the end of the PHARE-M session, and these posters were presented at the 1st CF Francophone Conference (2014). Three posters and their updates after 3 years were presented at the European CF conference (2012, 2014 and 2015) and the North American CF conference (2012). Videos featuring best practice recommendations concerning respiratory physiotherapy, physical and sports activities were prepared.

### Improvement of the patient registry

The French Registry contains one value in a given year for patient health outcomes and long-term treatments, while patient data are recorded at each clinic visit in the electronic patient record within the hospital information system. The Registry Committee establishes rules to select the clinic visit in a given year from which the FEV1, height and weight values are taken to be transmitted to the Registry.

In 2011–2012, the histograms presenting the median values of the centers remained anonymous in the Patient Registry report by center. The transparency brought in the PHARE-M meetings opened up discussions between the teams, leading them either to focus on the themes of improvement when the centers presented unsatisfactory results compared to national median values, or to question the measurement processes at the center. An on-site quality audit of the data transmitted to the Registry was organized in 2014–2015 pointed to variability in the measurement processes and in the application of the selection rule [28]. Avenues for improvement have been identified to support quality improvement of the data transmitted to the Registry by the centers.

To respond to the requests were made to the Registry team, the body mass index (BMI) for children was presented in Z-score value for LLC2. The lag between the year to which the data refer and the time of publication of the report (approximately 2 years in 2011) led the teams to supplement the Registry data with more recent data pulled directly from their patient records. The 2015 Patient Registry report has been issued by the end of 2016 and then provide more actual data for the PHARE-M LLC5.

### Sociological assessment of PHARE-M introduction

The assessment pointed to themes related to cultural acceptance of the PHARE-M at the time of its introduction:

1. the progressive adherence by the teams at the centers to the different steps of the program, taking into account initial feelings of resistance towards administrative hospital quality processes and the associated system of formalization. Putting patient outcomes at the different centers into perspective sparked interest in the process and clarified its purposes. The rapid consensus reached on the priority theme for improvement and the preparation of the poster were unifying;
2. the successful organization of the PHARE-M project, i.e. at national level (program coordinator and program management) and at local level (quality improvement team). However, on the local level, the specific difficulty and required skills of the “referent” position suggested that the role of the “referent” should not be taken by the physician in the quality improvement team and that the functioning of the physician leader/referent tandem is essential for the dynamic of the team.
3. the innovation consisting of patient or parent participation on the quality improvement teams, alongside their care providers, and their presence at the national face-to-face meetings as well as several local meetings was well perceived [29].
4. the gains for the functioning of the center teams were identified:

- a “collective enlisting of the team” for a unifying, energizing project for which the team learns to work together on what can be improved, thereby creating a “professional dynamic” in which professionals give new meanings to collective and profession-specific work practices;
- “reflexivity” on practices and relationships with patients/parents;
- a “calling into question” of care processes in front of other teams and transparency of outcomes, which may be sustained in a spirit of humility and desire to improve
- a “chance to speak” for all participants, which was possible in the melting pot of the face-to-face meetings;
- “rationale work” around the tools and processes, which objectivized and formalized practices and established a discourse to patients and parents;
- “dissemination” among the teams regarding quality management and tools;
- a “small-gains approach,” which allowed pragmatic actions to be implemented with often limited resources and outcomes to be measured to consolidate practices.

### The assessment for the patient organization funding recommendations

The consultant highlighted factors related to the feasibility and satisfaction regarding the PHARE-M training year:

- the 5P diagnosis phase faced challenges of feasibility within the training year with respect to 1) analysis of patient data, as Registry indicators were published with a two-year lag and BMI was expressed as an absolute value and not as a Z-score, and 2) analysis of patient satisfaction, as it took longer than expected for patients and parents to return their responses to the questionnaire*;*
- acceptance of the method was overall good, with the teams affirming that they were able to use the tools effectively and will be able to continue to do so beyond the training;
- team satisfaction was high concerning the consensus choice of a theme for improvement, the ability to comment on how they dealt with their work at sometimes difficult times (departures and reduced team), and the enlisting of the team around a joint project to improve patients’ outcomes; and
- implementing the actions at the centers met with several difficulties: the building of a consensus on the choice of priority and feasible actions, for example, therapeutic patient education, which does not always build a consensus on the teams; the availability of the resources to perform certain actions, for example, dieticians who cannot always be enlisted to abide by reconfigured care processes; cultural differences between teams that acted as obstacles to disseminating potential best practices.

Finally, the consultant assessed the effectiveness of the program according to the following criteria:

1. sustainable care improvement: **high,** due to adoption of perpetuated tools or practices;
2. improvement in patient health outcomes: **weak after 1 year**, except in a limited sample of patients included in the new process of care related to improvement actions;
3. development of professional expertise: **average**, especially when there was a slow start; and
4. development of a partnership with patients/parents and care providers: **limited** to the patients involved in the new process of care.

He concluded that PHARE-M mainly impacted care quality by allowing centers to use existing resources and innovative actions to comply with CF care recommendations, and that such an impact on quality of care should improve other aims, including the partnership with families and patients, provided that the patient organization support is strengthened.

### Clinic visit process redesign

**Fig. 1:**
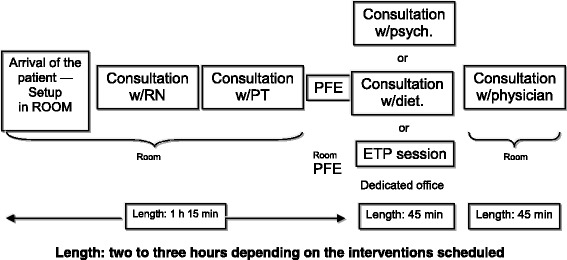
Example of multidisciplinary consultation process at a pediatric CFC

During the on-site coaching visits, the clinic visit process was analyzed by the program coach coordinator according to patient shadowing and process mapping. Multidisciplinary team (MDT) staff meetings, at which patients’ situations and treatment plans were determined, were also analyzed. Observation of the multidisciplinary consultation process enabled identification of seven key steps of an “optimal” process (Fig. 1) and description of the tasks corresponding to each step (Table 2).

**Table 2:** Description of the steps of the multidisciplinary consultation process

No.	Step	Who	What	Length (min)	Protocol
1	Installation of the patient	RN	- Setup in the dedicated room - Collection of new elements since the last visit - Verification of the results of examinations performed in the community or at the hospital - Needs for administrative documents (transport passes and certificates) - Reminder of the hygiene rules (wearing a mask) - Validation of the day’s clinic visit circuit	5–10	Hygiene — CR
2	Consultation w/nurse	RN	- Taking of measurements (weight and height) - Recording of the assessment in the patient’s electronic record - Taking stock of the treatments prescribed and taken - Care (implantable device, blood draw, etc.) - Events in the life of the patient to be prepared - Responses to the patient’s/parent’s questions	20–30	Measurement protocol (height and weight) according to the patient’s age
3	Respiratory assessment	PT	- Implementation of the hygiene protocol - Taking stock of the physiotherapy practiced in the community and review of instrumental aids - Taking stock of physical and sports activities - Physiotherapy session with sputum collection for sputum culture - Assessment of bronchial congestion - Recording of the assessment in the patient’s electronic record	40	
4	PFT (pulmonary function test)		- Measurement of respiratory function - Recording in the patient’s electronic record	10	Recommendations of the American Thoracic Society
5	Other scheduled intervention		- Psychological assessment (psychologist), social assessment (social worker), or nutritional assessment (dietician) - Or individual therapeutic education session - Recording of the assessment in the patient’s electronic record	30–40	
6	Medical consultation	Physician	- Additional examination - Clinical examination - Review of all treatment - Response to the patient’s/parent’s questions - Referral to the referent professional - Planning of the next visit and need for additional examinations to be performed at the hospital or in the community - Preparation of prescriptions - Recording in the patient’s electronic record - Signing of medical certificates	35–45	End of the course of consultation to benefit from assessments performed by the other professionals recorded in the patient’s electronic record
7	Departure of the patient	Admin. Sec. or RN	- Scheduling of the next appointment - Review of organization for departure (transport, nutritional need, and support) - Verification that the patient has all useful documents - Instructions for events by the next visit - Once the patient leaves the room, disinfection before accommodating the next patient.	30	Disinfection protocol

Implementation of the process first of all depends on the configuration of spaces. It also incorporates a therapeutic patient education session into the visit. It is linked to multidisciplinary staff meeting at which team members exchange information and hold discussions to ensure that the patient receives genuinely interdisciplinary care and that essential organizational aims are achieved: i) anticipating the consultations scheduled for the following week and having the professionals confirm their planning for these visits by specifying their aims for the patient; ii) drawing conclusions on the situation of the patients seen in the past week and establishing actions to be coordinated before the next visit by the professional in charge of monitoring them; and iii) preparing the visit report and scheduling the next visit.

Most coaching visits pointed out difficulties in sticking to this optimal process. At several centers, there was not enough time to review the situation of all patients seen the past week; as a solution to this problem patients having had an Annual Review or patients with specific needs were prioritized. It was sometimes difficult to get the entire MDT to meet at the same time. Patient records could not always be displayed during the staff meeting. Time was wasted on sharing data rather than making decisions. Effective meeting skills were developed and actions were taken according to a Professional Practice Evaluation process in order to improve the clinic visit process and the staff meeting.

### PHARE-M standardization into a CPD program

The PHARE-M was approved as a multidisciplinary CPD program in 2014, and the 2015 PHARE-M LLC3 could be offered as a CPD program with the following features:

1. The PHARE-M as a CPD program received the approval of the Medical and Paramedical Independent Scientific Committees and will be re-evaluated prior to the extension of this approval (2021); formalized evaluation of each PHARE-M annual session is the responsibility of the hospital continuous education authority.
2. The training center at the Roscoff Foundation runs the PHARE-M CPD program, and the teams’ registration fees provide the national team resources to continue to assess, improve and up-date the program and its website.
3. An annual request for application from the director of the Roscoff Center of Expertise, sent in May, invites and reminds the centers to register for the PHARE-M on a volunteer basis; an information meeting is organized in October to present the program and provide documentation to hospital continuing education directorates and quality departments.
4. The professionals on the team at the centers take administrative steps at their hospital to apply for the multidisciplinary PHARE-M CPD program to register for the next year and earn further CPD credits; the professionals on the CF team who are registered must include a lead physician lead and four to five multidisciplinary professionals.
5. The professionals on the teams at the centers are authorized to be absent from their posts for CPD training meetings, both face-to-face and web meetings, and another professional should replace them in their absence.
6. The professionals on the teams at the centers are reimbursed for their travel fees by hospital continuing education.
7. The patient organization is asked to reimburse the travel fees of the patients/parents and for the professionals unable to register to the PHARE-M CPD program.
8. The patient organization is continuing to fund 0.20 FTEs for the extra-time required for a PHARE-M referent on each team during the training year.

## Discussion

The PHARE-M represented a “complex intervention” in clinical microsystems embedded in hospital systems marked by their diversity, their constant evolution, and the current economic pressure on the health care system. The various aspects of the program, essentially putting patient outcomes at the heart of quality improvement efforts and involving patients and parents on the quality improvement teams, led to a rapid consensus on the priority theme for improvement and identification of improvements on the process of care. Barriers linked to cultural differences between the United States and France were overcome by “Frenchifying” the Action Guide and the training material. This went beyond translating them into French, and involved searching for synergies with the quality departments. The PHARE-M contributed to the hospital certification process, and thanks to hospital continuing education reform, it was recognized as a multidisciplinary CPD program.

### Limitations of the program roll-out

The pace of the roll-out of the PHARE-M throughout France could be accelerated by identifying sources of leverages. This would require professionals and patient organization representatives to pool their efforts (Table 3).

**Table 3:** Next steps to accelerate the pace of the roll-out of the PHARE-M in France

***1 Develop the French*** **CF** ***Registry*** - Reduce the time taken to produce annual Registry reports; - Achieve public transparency of the results by center; - Advance towards an encounter-based national CF database which produces annual Registry reports as well as ongoing (quarterly) results for the monitoring of the QIPs at the centers
***2 Strengthen the motivation of the teams to enroll in PHARE-M program*** - Report the PHARE-M experience, results and satisfaction during professional conferences and patient organization assemblies; - Get the CF community leadership, professionals and the patient organization more involved in continuous quality improvement; - Continue to obtain funding from the patient organization for the extra-time needed for the PHARE-M referent at each center during the training year; - Validate continuing professional development credits through the PHARE-M; - Maintain a focus on continuous quality improvement with financial support for post-PHARE-M cycles until other funding is available (see below); - Develop a convergence between the roll-out of the PHARE-M and other actions to increase the availability of professional resources, access to CF care guidelines translated in French, and tutoring by discipline within the network;
***3 Consolidate and develop expertise and resources for the PHARE-M*** - Organize a community of PHARE-M referents from the centers for advanced training on measurement, effective meeting skills, quality tools (fishbone diagrams, PDSAs, patient shadowing); - Develop a culture of publishing QI initiatives according to SQUIRE standards - Improve and adapt the PHARE-M website to show the various aspects of the program (registration to the CPD program, international research, international community ties, publications, etc.…)
***4 Build alliances at the hospital and national health system levels*** - Continue contributing to the hospital certification process, supporting the hospital quality department through improvement actions, Professional Practice Evaluations, or hospital quality indicators; - Develop new CPD programs for post PHARE-M cycles focusing on providing reminders of the QI method and tools, benchmarking, measuring and writing for publications; - Participate in conferences of health authorities or working groups aimed at care quality improvement and patient involvement in healthcare to promote this QI LLC method;

### Factors for success in replicating the US CF LLC program

#### Developing an understanding of the initial model of improvement…

The 2006 Dartmouth and CF LLC model included involving patient and family on CFC improvement teams, using standardized evidence and practice-based ideas for change, preparing regular CF center progress reports, coaching teams, actively using the Patient Registry and applied measurement, and getting to know patients and families through observation and inquiry skills [6]. The following actions laid the foundations for an in-depth understanding of the method and its effects and dynamics: training the physician leader, the physiotherapist and the parent engineer on the national team at the Dartmouth Institute, giving them the opportunity to closely observe US CFCs with a long history of engagement in LLCs, increasing their awareness and energizing them through participation in several US LLC face-to-face meetings at the annual North American CF Conference, and training the parent to the “Coach the coaches” course. The method cannot be learned in its entirety from books, and the practical experiences of the US centers were enlightening. The supervision of the translation by the Dartmouth Institute and the CFF ensured that the training material initially conformed to the improvement model. The humility of the national team, who recongnized its inability to understand the whole QI approach in depth through training and visits to centers alone, led them to stick to the US Action Guide and training materials during the French LLC1.

#### … and then adapting the model to the French context

Inevitably, the first LLC had to face the cultural gap between the US and France. This would have led to a great deal of conflict had the national team not anticipated cultural shock and asked the teams to help adapt the program to the French context. Opening up this opportunity decreased the tensions which arose as much from the program as they did from existing frustrations towards the hospital system: burdensome administrative quality procedures, economic pressure on the teams, inadequate facilities, and insufficient resources in every discipline in the CF team compared to standards of care were some of the issues that made the teams uncomfortable with the program.

The modifications made to LLC2 consisted mainly of replacing examples from US teams with examples from French pilot teams in the Action Guide and simplifying some of the theoretical presentations that the pilot teams had rejected, such as the reminders of QI in industry (e.g., process optimization steps) and statistical measurement techniques (e.g., control limits). On-site coaching was intensified and focused on patient shadowing and process mapping, which appeared to be more relevant and usable for the teams. After 3 years, as the teams engaged in LLC1 and LLC2 were invited to report their results, measurement became a new priority. This topic was addressed in post PHARE-M cycles while writing for publication was envisaged and SQUIRE guidelines were presented.

#### Performativity of the process initiated with the PHARE-M

All processes pertaining to care quality are evaluated and judged by the professionals with respect to their performativity, that is to say, their contribution by acts that bring about the reality uttered by this process. The notion of “performativity,” borrowed from linguistic pragmatics, shows that the medical and healthcare sciences in particular, in the case examined here, and the sciences in general, are not limited to representing the world: they also make it, cause it, and form it, at least to a certain extent and under certain conditions. In linguistics, an utterance is said to be performative when it establishes that of which it speaks. Extended and adapted to the sciences, this insight allows the classification of situations in which the subject of a methodological work is not merely observed or described, but modified or even called into being. “When the players started to prepare and produce their data and their poster, to exchange and compare experiences, the performative capacity of the PHARE-M was perceived and legitimized. The performativity of the action guide was revealed and rationalized in the eyes of the participants on the teams after a few months, when the results that they had presented and debated highlighted the method's organizing nature”. The salience of the outcomes that are put in perspective, the feeling of having reinvested in care tasks, and the perception of producing and thinking differently most precisely characterize the program’s performance. The medical and healthcare population generally had a negative conception of the quality engineering movement. Its culture is the very opposite of the medical, clinical, and healthcare culture which, from the outset, conceives of quality as something incorporated into individual practice, not something existing outside of individual practice or tied to an organization. PHARE-M partially reconciled these two visions.

#### On-site coaching

The recommendation concerning the strengthening of on-site coaching was verified to be operative during LLC2, with the establishment of visits by the coach coordinator, which at once allowed process mapping to be performed and organizational problems to be addressed. Team coaching was underlined as the most effective measure to develop the capability for improvement of the multidisciplinary teams at the centers [6]. However, this undertaking is costly and could not be offered to the centers during LLC1, as no specific funding had been obtained from the patient organization. Following the assessment, some funding was offered for LLC2 through a specific grant from the Foundation ildys. This grant acted as an investment in the future development of the PHARE-M as a CPD program supported by the training center at the foundation: on-site coaching could be offered, but not at the level achieved in the US. To compensate for the lack of on-site coaching, it was decided to develop the skills of one member of each CF team, referred to as the PHARE-M referent, and to search for synergy with the hospital quality department.

#### Synergy between therapeutic patient education and patient/parent involvement in QI

Therapeutic patient education in cystic fibrosis has been developed in French CF care, especially at pediatric centers, as it was recognized by law in 2009 as a right for persons suffering from chronic diseases. In practice, it establishes a lasting alliance between the healthcare team and the patient/parent with a view to developing the latter’s autonomy and adaptation skills, adjusting them regularly as their needs evolve, and working to remove obstacles to establishing treatments [30]. On the PHARE-M side, the national team fostered patient and parent involvement as a pre-requisite for participation in the program, integrating them as members in the quality improvement team at their center as members so that they would contribute the user’s point of view to QI and potentially co-design care processes [31, 32]. This convergence between the two dimensions of patient involvement, in self-care and in the process of care redesign, was innovative in 2011 in France, based on the experience of the national team experience rather than on science.

More specifically, the national team fostered links between care improvement actions and educational interventions during the care process. The participation of the patients/parents on the quality improvement teams made it possible to ensure that their preferences and experiences were taken into account when new processes were proposed or care was intensified (nutritional care). Furthermore, within the framework of the PHARE-M, therapeutic education actions were strengthened as sources of leverage to improve home care and thus improve patient outcomes. Prioritizing certain health aims led to priority education actions. Reorganizing multidisciplinary clinic visits allowed an educational session to be incorporated into the course of the visit. Sharing of educational tools among the teams participating in the PHARE-M was boosted. A tool to identify and react to pulmonary exacerbations (REACT) was developed by the national TPE working group after the teams identified the variability in the practices of diagnosing and treating pulmonary exacerbations. Despite fears of therapeutic education competing for space in the teams’ tight schedules, the PHARE-M strengthened the practice of PTE and the use of educational tools.

## Conclusions

### Prospects for the roll-out of PHARE-M and a CQI process in CF care in France

As of early 2017, the PHARE-M has been implemented at 23 centers (out of 45) and LLC6 is ongoing with adult teams. The teams’ satisfaction is still increasing, with a median score of 9.1 for LLC5, which was a pediatric program. The outcomes of the centers will be made transparent among the professionals and the patient organization board only in the next few months. Public transparency will take more time.

The research program is aimed at assessing the impact of the PHARE-M on patient outcomes after 3 years, though it may be difficult to establish a causal link to the PHARE-M, given the evolving context in which centers operates and CF treatments are provided, and the bias inherent to recruiting centers that volunteer to participate. The realistic assessment will conduct an in-depth examination of “how and why” a stronger impact of the PHARE-M may have been observed at certain centers engaged in PHARE-M [33]. Presenting the results of the research program in 2017 and publishing on PHARE-M initiative will definitely increase the visibility of PHARE-M and raise awareness in France on this quality improvement approach.

Six years after the PHARE-M was launched in the CF network in France, half the centers have been trained, and the various stakeholders – professionals, patient organization representatives and hospital quality department members in some hospitals – perceive the strength of this LLC QI approach and wish to participate in it and contribute to rolling it out further. Interest in this approach is growing outside of CF care, for example among hospital quality professionals willing to test patient shadowing in other chronic care departments. Beyond these short-term contributions, the need for overall reflection to adapt the method to another model of care (translated in a disease specific Action Guide) requires a dedicated task force at an appropriate level of the health system. Experience with the QIP in CF may inspire its application to the care of other chronic diseases, and this article may contribute to its dissemination.
